# Medicaid Expansion and Mortality Among Persons Who Were Formerly Incarcerated

**DOI:** 10.1001/jamanetworkopen.2024.29454

**Published:** 2024-09-17

**Authors:** Pasangi S. Perera, Vanessa E. Miller, Kate Vinita Fitch, Monica E. Swilley-Martinez, David L. Rosen, Lauren Brinkley-Rubinstein, Brandon D. L. Marshall, Brian W. Pence, Andrew L. Kavee, Scott K. Proescholdbell, Rosemarie A. Martin, Lewis J. Peiper, Shabbar I. Ranapurwala

**Affiliations:** 1 Department of Epidemiology, Gillings School of Global Public Health, University of North Carolina, Chapel Hill; 2 Injury Prevention Research Center, University of North Carolina, Chapel Hill; 3 Department of Obstetrics and Gynecology, School of Medicine, University of North Carolina, Chapel Hill; 4 Division of Infectious Diseases, School of Medicine, University of North Carolina, Chapel Hill; 5 Department of Population Health Sciences, Duke University School of Medicine, Durham, North Carolina; 6 Department of Epidemiology, Brown University School of Public Health, Providence, Rhode Island; 7 The Cecil G. Sheps Center for Health Services Research, University of North Carolina, Chapel Hill; 8 Injury and Violence Prevention Branch, North Carolina Department of Health and Human Services, Raleigh; 9 Department of Behavioral and Social Sciences, Brown University School of Public Health, Providence, Rhode Island; 10 Division of Comprehensive Health Services, North Carolina Department of Adult Correction, Raleigh

## Abstract

**Importance:**

Since 2014, Medicaid expansion has been implemented in many states across the US, increasing health care access among vulnerable populations, including formerly incarcerated people who experience higher mortality rates than the general population.

**Objective:**

To examine population-level association of Medicaid expansion with postrelease mortality from all causes, unintentional drug overdoses, opioid overdoses, polydrug overdoses, suicides, and homicides among formerly incarcerated people in Rhode Island (RI), which expanded Medicaid, compared with North Carolina (NC), which did not expand Medicaid during the study period.

**Design, Setting, and Participants:**

A cohort study was conducted using incarceration release data from January 1, 2009, to December 31, 2018, linked to death records from January 1, 2009, to December 31, 2019, on individuals released from incarceration in RI and NC. Data analysis was performed from August 20, 2022, to February 15, 2024. Participants included those aged 18 years or older who were released from incarceration. Individuals who were temporarily held during ongoing judicial proceedings, died during incarceration, or not released from incarceration during the study period were excluded.

**Exposure:**

Full Medicaid expansion in RI effective January 1, 2014.

**Main Outcomes and Measures:**

Mortality from all causes, unintentional drug overdoses, unintentional opioid and polydrug overdoses, suicides, and homicides.

**Results:**

Between 2009 and 2018, 17 824 individuals were released from RI prisons (mean [SD] age, 38.39 [10.85] years; 31 512 [89.1%] male) and 160 861 were released from NC prisons (mean [SD] age, 38.28 [10.84] years; 209 021 [87.5%] male). Compared with NC, people who were formerly incarcerated in RI experienced a sustained decrease of 72 per 100 000 person-years (95% CI, −108 to −36 per 100 000 person-years) in all-cause mortality per quarter after Medicaid expansion. Similar decreases were observed in RI in drug overdose deaths (−172 per 100 000 person-years per 6 months; 95% CI, −226 to −117 per 100 000 person-years), including opioid and polydrug overdoses, and homicide deaths (−23 per 100 000 person-years per year; 95% CI, −50 to 4 per 100 000 person-years) after Medicaid expansion. Suicide mortality did not change after Medicaid expansion. After Medicaid expansion in RI, non-Hispanic White individuals experienced 3 times greater sustained decreases in all-cause mortality than all racially minoritized individuals combined, while non-Hispanic Black individuals did not experience any substantial benefits. There was no modification by sex. Individuals aged 30 years or older experienced greater all-cause mortality reduction after Medicaid expansion than those younger than 30 years.

**Conclusions and Relevance:**

Medicaid expansion in RI was associated with a decrease in all-cause, overdose, and homicide mortality among formerly incarcerated people. However, these decreases were most observed among White individuals, while racially minoritized individuals received little to no benefits in the studied outcomes.

## Key Points

**Question:**

Was Medicaid expansion associated with changes in mortality rates among formerly incarcerated individuals in Rhode Island?

**Findings:**

In this cohort study, after Medicaid expansion, 238 781 formerly incarcerated persons in Rhode Island experienced sustained decreases in mortality rates from all causes; drug, opioid, and polydrug overdose; and homicide; suicide mortality did not change. However, these decreases were largely experienced by White individuals, while racially minoritized individuals experienced little to no decreases in mortality rates.

**Meaning:**

The findings of this study suggest that Medicaid expansion may result in a reduction of mortality rates among formerly incarcerated people; however, there appear to be racialized inequities in the impact of Medicaid expansion.

## Teaser

This cohort study compares mortality rates before and after Medicaid expansion in individuals who were formerly incarcerated.

## Introduction

People with a history of incarceration are at higher risk of mortality than their general population peers, especially from drug overdose, homicide, and suicide.^1,2,3,4,5,6,7,8^ After release from incarceration, people face stigma and lack access to housing, employment, and health care, which increase the risk of poor health outcomes, including drug overdose and violent deaths from suicide and homicide.^1,2,4,5,6,7,8,9,10^ People who were formerly incarcerated are disproportionately Black, Hispanic, and socioeconomically disadvantaged, which exacerbates racialized health inequities.^2,6,7^

Medicaid expansion, first effective on January 1, 2014, in 25 states and now adopted in 40 states and Washington, DC, could reduce mortality among people who were formerly incarcerated by increasing access to medications for opioid use disorder (MOUD), mental health care, and health care.^11,12,13,14,15,16,17^ The US Government Accountability Office found that the expansion of Medicaid under the Affordable Care Act made health care available to 80% to 90% of people who were formerly incarcerated who might have been previously ineligible in those states.^11^ However, to our knowledge, the outcomes of Medicaid expansion have not been evaluated. One study examining the impact of prerelease Medicaid enrollment in Wisconsin, an expansion state, found that OUD care outpatient visits increased by 9.24% (95% CI, 7.46%-11.02%) among White individuals, but by only 5.28% (95% CI, 3.34%-7.22%) among Black individuals.^18^ A study from Rhode Island (RI) reported that, following implementation of a statewide program to provide MOUD in correctional facilities, the opioid overdose death rate decreased by 60.5% among persons who were formerly incarcerated, from 2016 (pre) to 2017 (post), but this decrease was experienced by White individuals only.^16^ Since individuals who were formerly incarcerated disproportionately belong to racially minoritized and socioeconomically disadvantaged groups, and these prior studies point to racialized inequities, it is important to examine the outcomes of Medicaid expansion by racialized groups.

In this study, we evaluated the association of Medicaid expansion with postrelease all-cause, drug overdose, homicide, and suicide mortality among people who were formerly incarcerated and examined racialized, age-based, and sex-based disparities within this association. We used a robust, quasi-experimental design, leveraging a natural experiment due to the implementation of Medicaid, and compare time trends of the outcome in RI that fully expanded Medicaid on January 1, 2014, with North Carolina (NC), which had not expanded Medicaid during the study period.

## Methods

In this quasi-experimental cohort study from 2009 to 2019, we conducted controlled interrupted time series analysis to evaluate immediate and sustained trend changes in postrelease mortality rates among persons who were formerly incarcerated due to Medicaid expansion in RI, which fully expanded Medicaid on January 1, 2014, compared with NC, which had not expanded Medicaid during the study period.^19,20,21,22,23^ The study was approved by the University of North Carolina at Chapel Hill Institutional Review Board, which provided a waiver for informed consent. Data were deidentified. This study followed the Strengthening the Reporting of Observational Studies in Epidemiology (STROBE) reporting guideline.

### Setting and Participants

Incarceration release data from the RI Department of Corrections and NC Department of Adult Corrections from January 1, 2009, to December 31, 2018, were linked to each state’s death certificate data from January 1, 2009, to December 31, 2019, to allow at least 1 year of follow-up for each person released from incarceration. The data were linked deterministically using Soundex of last name, Soundex of first name, date of birth, and sex. We linked NC data at the University of North Carolina, while the RI data were linked at the RI Department of Health, using the same method, then shared deidentified data. The incarceration release data included release date, incarceration date, age at release, self-reported race and ethnicity, and sex. Release records were excluded if a person was being temporarily held while the judicial proceedings were ongoing (as they may not have been convicted and may have a different experience than those with convictions and incarcerations), younger than 18 years at release, died while incarcerated, or was not released from incarceration before the end of study period. Postrelease person-time was calculated as time in the community from the date of release until reincarceration, death, or the end of the study, whichever came first. Individuals who experienced reincarceration contributed multiple periods of person-time upon release throughout the study period.

### Outcomes

We examined all-cause mortality and deaths due to unintentional drug overdose, unintentional opioid and polydrug overdose, suicide, and homicide, defined using death certificate *International Statistical Classification of Diseases and Related Health Problems, 10th Revision* codes (eTable 1 in Supplement 1). One-year postrelease mortality rates were calculated for each outcome by dividing the number of deaths within 365 days of release by the person-years accrued within 365 days post release. Two-year postrelease homicide and suicide rates were calculated in the same manner. We calculated 2-year postrelease homicide and suicide death rates instead of 1-year postrelease death rates because both are rare outcomes and the RI formerly incarcerated population was small, limiting the ability to calculate stable 1-year postrelease mortality rates. We created separate time series of these 1- and 2-year mortality rates using quarterly, 6-month, and annual intervals from 2009 to 2019 for both the RI and NC formerly incarcerated populations. All rates are expressed per 100 000 person-years.

### Exposure

We used the Medicaid expansion implementation date of January 1, 2014, in RI as the interruption in the controlled interrupted time series analyses, such that January 2014 was the first postintervention month (lagged effects are addressed in the Statistical Analysis section). North Carolina, a nonexpansion state, served as a control for natural trend changes, national policy landscape, and seasonality in mortality rates among people who were formerly incarcerated. Our analyses assumed that NC represents a reasonable counterfactual of what the mortality rates in RI would have been without Medicaid expansion.

### Covariates

Self-reported race and ethnicity are available as Hispanic of any race, non-Hispanic American Native, non-Hispanic Asian or Pacific Islander, non-Hispanic Black (hereinafter, Black), non-Hispanic White (hereinafter, White), or multiple race/unknown. Sex was categorized as male, female, or unknown. To examine effect measure modification (EMM) while maintaining statistically powered group sizes, race and ethnicity was collapsed as Black, White, or combined group of all racially minoritized individuals. Age was collapsed as younger than 30 years and 30 years or older.

### Statistical Analysis

We conducted controlled interrupted time series analysis using autoregressive integrated moving average models to compare mortality rates among people who were formerly incarcerated in RI (intervention state) with NC (nonintervention/control state) before and after the implementation of Medicaid expansion in RI in 2014.^19,20,21,22,23^ The statistical model is presented in eMethods 1 in Supplement 1.

From the autoregressive integrated moving average model (eMethods 1 in Supplement 1), we evaluated both immediate level changes (β_6_), representing absolute outcome rate change immediately (ie, the quarter, 6-month, or year period) after the policy implementation and sustained trend changes (β_7_), representing the change in trend of the outcome rate after policy implementation as well as 95% CIs. We examined EMM by race and ethnicity and age group to estimate potential differential effects of Medicaid expansion. We did not examine EMM by sex, given the small number of women formerly incarcerated in RI.

While Medicaid expansion was implemented in RI on January 1, 2014, access to care and subsequent improvements in public health are expected to improve slowly over time. Hence, we conducted sensitivity analyses by exploring time-lagged outcomes of the policy implementation date from January 2014 to later dates (eMethods 2 in Supplement 1). Since Medicaid expansion may impact reincarceration, we analyzed Medicaid expansion and reincarceration rates and if the Medicaid expansion and mortality association varied among people with only 1 vs multiple incarceration. Data analysis was performed from August 20, 2022, to February 15, 2024, using SAS, version 9.4 (SAS Institute Inc).

## Results

Between 2009 and 2018, 35 350 releases from RI prisons among 17 824 unique individuals (mean [SD] age, 38.39 [10.85] years; 31 512 [89.1%] male; 3838 [10.9%] female) met the inclusion criteria. In NC, 160 861 eligible individuals (mean [SD] age, 38.28 [10.84] years; 209 021 [87.5%] male; 29 760 [12.5%] female) were released, totaling 238 781 releases during the study period. Distributions of age at release and sex among people who were formerly incarcerated in RI and NC were similar in both pre- and post-Medicaid expansion (eTable 2 in Supplement 1). In both states, individuals who were formerly incarcerated disproportionately included racially minoritized people. However, NC had a higher proportion of individuals formerly incarcerated who were Black (50.8%) compared with RI (25.2%), and RI had higher proportions of individuals formerly incarcerated who were Hispanic (18.6%) and White (53.6%), compared with NC (Hispanic: 1.9% and White: 42.8%). The distribution of racialized groups did not change substantively before and after January 2014 (Medicaid expansion in RI) in both states (eTable 2 in Supplement 1). Rhode Island had higher mortality rates for all outcomes compared with NC except homicide (Table 1).

**Table 1.** Overall Death Rates per 100 000 Person-Years Among People Who Were Formerly Incarcerated in Rhode Island and North Carolina

Outcome	Rate (95% CI)					
	Overall, 2009-2018		Pre-Medicaid expansion, 2009-2013		Post-Medicaid expansion, 2014-2018	
	RI	NC	RI	NC	RI	NC
All-cause mortality rate, 1-y postrelease mortality rate	1069 (951-1197)	839 (801-879)	927 (779-1095)	660 (614-708)	1230 (1047-1435)	1058 (994-1125)
Unintentional drug overdose, 1-y postrelease mortality rate	583 (497-679)	251 (230-273)	437 (337-557)	129 (109-151)	749 (608-913)	401 (362-443)
Unintentional opioid overdose, 1-y postrelease mortality rate	522 (441-614)	217 (198-238)	390 (296-504)	99 (82-118)	672 (539-828)	362 (325-402)
Unintentional polydrug overdose, 2-y postrelease mortality rate	315 (252-388)	108 (95-123)	262 (186-358)	34 (24-46)	374 (277-495)	198 (171-229)
Suicide, 2-y postrelease mortality rate	79 (49-119)	39 (31-48)	87 (47-149)	32 (23-44)	69 (31-131)	47 (34-63)
Homicide, 2-y postrelease mortality rate	75 (46-115)	139 (124-156)	67 (32-124)	124 (105-146)	84 (42-150)	157 (133-184)

Abbreviations: NC, North Carolina; RI, Rhode Island.

### All-Cause Mortality

**Figure 1.**
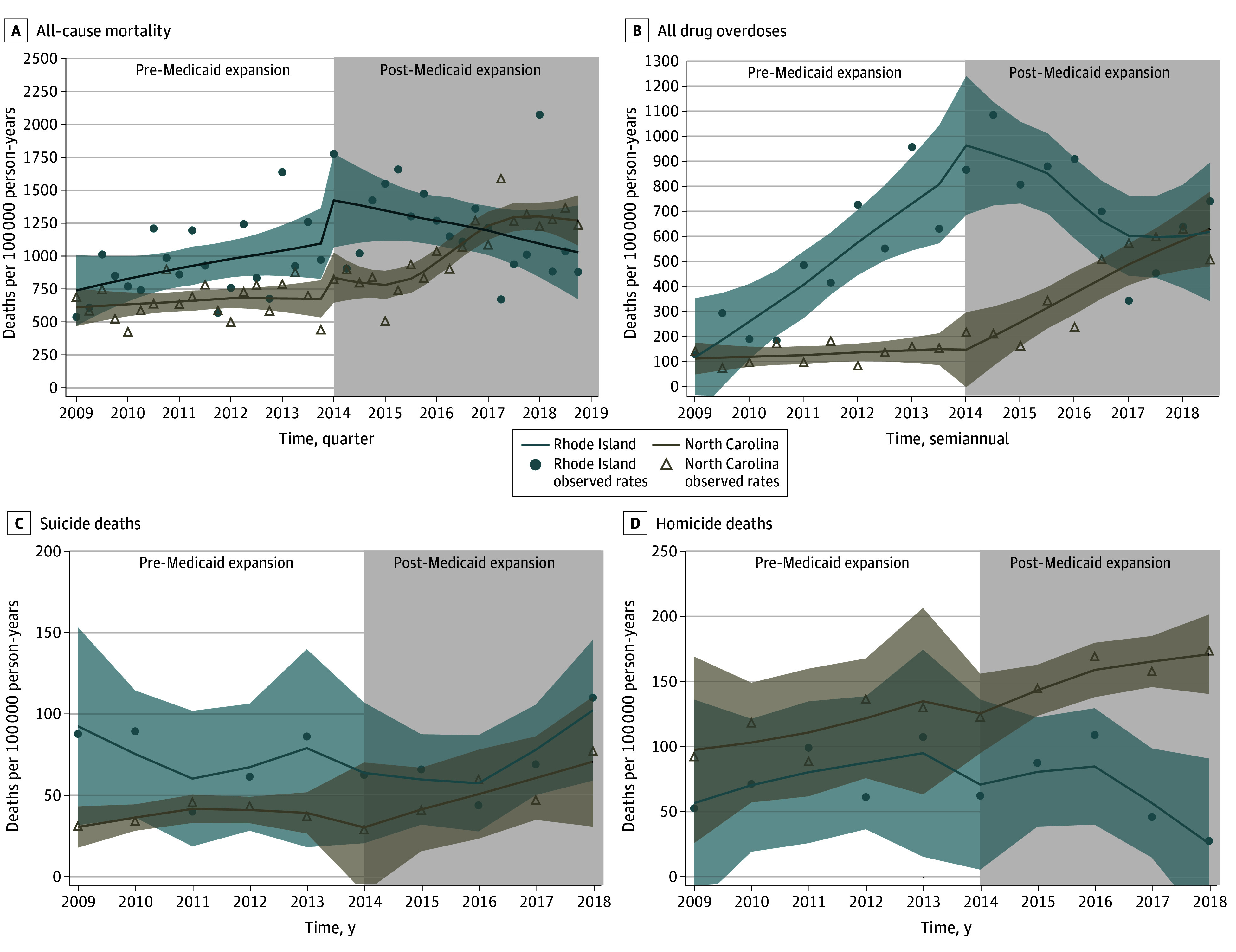
Association of Medicaid Expansion Implementation in 2014 in Rhode Island With All-Cause and Cause-Specific Mortality Compared With North Carolina, 2009-2019 Shaded areas indicate 95% CIs.

All-cause mortality rates among formerly incarcerated persons displayed increasing trends in both RI and NC before the RI Medicaid expansion; however, after Medicaid expansion, all-cause mortality decreased in RI, while NC rates continued to increase. Compared with NC, RI experienced a sustained decrease of 72 deaths per 100 000 person-years (95% CI, −108 to −36 per 100 000 person-years) among releasees each quarter after Medicaid expansion (Table 2; Figure 1A). This quarterly decrease amounted to about 289 fewer deaths per 100 000 person-years in RI in the first year after Medicaid expansion. Observed all-cause mortality rates in RI returned to the 2009-2010 levels by the end of 2019.

**Table 2.** Immediate and Sustained Changes in Mortality Rates After Medicaid Expansion Implementation in 2014 in Rhode Island Compared With North Carolina, 2009-2018

Outcome	Change in mortality rate after Medicaid expansion, β_6_ (95% CI)	
	Immediate absolute	Sustained trend
All-cause mortality^a^^,^^b^	280 (−135 to 695)	−72 (−108 to −36)
Unintentional drug overdose^a^^,^^c^	74 (−240 to 388)	−172 (−226 to −117)
Unintentional opioid overdose^a^^,^^c^	45 (−226 to 317)	−157 (−204 to −110)
Unintentional polydrug overdose^a^^,^^c^	−155 (−350 to 40)	−62 (−96 to −28)
Suicide^d^^,^^e^	1 (−61 to 64)	5 (−17 to 26)
Homicide^d^^,^^e^	−9 (−88 to 70)	−23 (−50 to 4)

^a^ One-year death rate per 100 000 person-years.

^b^ Trends calculated per quarter.

^c^ Trends calculated per 6-month intervals.

^d^ Two-year death rate per 100 000 person-years.

^e^ Trends calculated per year.

The all-cause mortality rates among White individuals were highest (eTable 3 in Supplement 1) and twice those in all racially minoritized groups combined (eTable 4 in Supplement 1). After Medicaid expansion, White individuals who were formerly incarcerated in RI experienced a sustained decrease of 388 deaths per 100 000 person-years (95% CI, −650 to −126 per 100 000 person-years) per year. This all-cause mortality change experienced by White individuals was 3 times that experienced by all racially minoritized individuals (−137; 95% CI, −399 to 125 per 100 000 person-years), and Black individuals did not experience any substantial change (−48; 95% CI, −317 to 221 per 100 000 person-years) (Table 3; eFigure 1 in Supplement 1).

**Table 3.** Immediate and Sustained Changes in Mortality Rates After Medicaid Expansion Implementation in 2014 in Rhode Island Compared With North Carolina by Race and Ethnicity and Age, 2009-2018^a^

Characteristic	Change in mortality rates after Medicaid expansion (95% CI)					
	All-cause mortality^b^		Unintentional drug overdose^b^		Unintentional opioid overdose^b^	
	Immediate, β_6_	Sustained, β_7_	Immediate, β_6_	Sustained, β_7_	Immediate, β_6_	Sustained, β_7_
Race and ethnicity						
Non-Hispanic Black	−396 (−1181 to 388)	−48 (−317 to 221)	NE	NE	NE	NE
Non-Hispanic White	342 (−422 to 1106)	−388 (−650 to −126)	136 (−539 to 810)	−516 (−747 to −284)	6 (−610 to 622)	−455 (−666 to −244)
All racially minoritized groups combined	−77 (−841 to 687)	−137 (−399 to 125)	−165 (−572 to 242)	−166 (−306 to −26)	−67 (−403 to 268)	−167 (−282 to −52)
Age, y						
<30	593 (−299 to 1485)	−174 (−480 to 132)	304 (−230 to 839)	−228 (−411 to −44)	21 (−270 to 312)^c^	−111 (−210 to −11)^c^
≥30	−207 (−706 to 292)	−357 (−528 to −186)	−261 (−790 to 269)	−448 (−630 to −266)	−101 (−404 to 203)^c^	−285 (−389 to −181)^c^

Abbreviation: NE, not estimable due to small population.

^a^ All trends calculated per year using 1-year death rates per 100 000 person-years unless indicated otherwise.

^b^ A complete explanation of the model and model parameters is available in eMethods 1 of Supplement 1.

^c^ Two-year death rate per 100 000 person-years.

The all-cause mortality rate among individuals aged 30 years or older was twice the rate among individuals younger than 30 years (eTable 4 in Supplement 1). Those aged 30 years or older experienced a sustained decrease in all-cause mortality rates after Medicaid expansion that was twice as large as those among individuals younger than 30 years (Table 3; eFigure 4 in Supplement 1).

### Unintentional Drug, Opioid, and Polydrug Overdose Deaths

Unintentional overdose mortality rates increased in both RI and NC prior to Medicaid expansion, but increased more rapidly in RI (Table 1, Figure 1B). After Medicaid expansion, compared with NC, people who were formerly incarcerated in RI experienced a sustained decrease of 172 deaths per 100 000 person-years (95% CI, −226 to −117 per 100 000 person-years) per 6-month interval (Table 2; Figure 1B).

Unintentional overdose mortality rates were highest in White individuals and almost twice the rates among racially minoritized groups combined (eTables 3 and 4 in Supplement 1). After Medicaid expansion implementation in RI, White individuals underwent a large, sustained decrease in mortality of 516 deaths per 100 000 person-years (95% CI, −747 to −284 per 100 000 person-years) per year compared with racially minoritized individuals, who experienced a sustained decrease in mortality of 166 deaths per 100 000 person-years (95% CI, −306 to −27 per 100 000 person-years) per year (Table 3; eFigure 2 in Supplement 1).

Individuals aged 30 years or older had higher drug overdose mortality rates compared with individuals younger than 30 years. After Medicaid expansion in RI, individuals aged 30 years or older experienced 2 times greater sustained decrease in mortality rates per year than those younger than 30 years (Table 3, eFigure 5 in Supplement 1).

**Figure 2.**
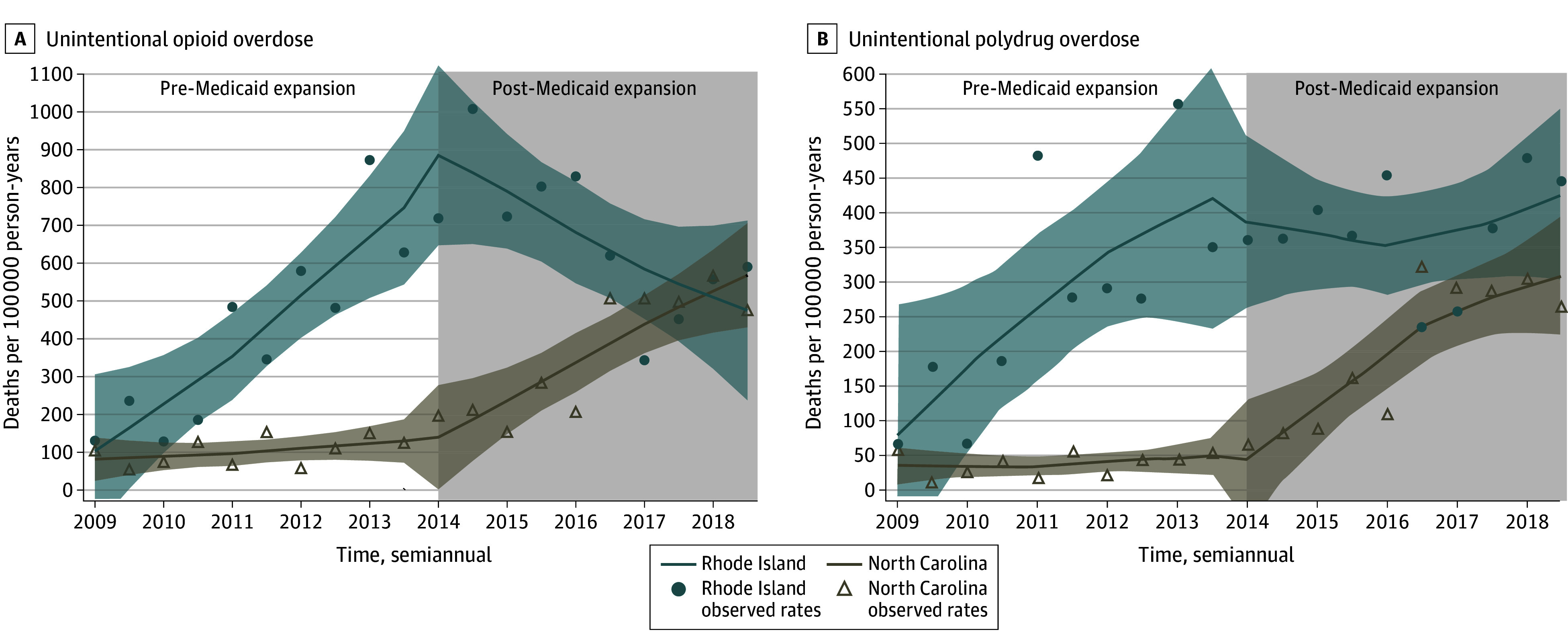
Association of Medicaid Expansion Implementation in 2014 in Rhode Island With Unintentional Opioid and Polydrug Overdose Mortality Compared With North Carolina, 2009-2018 Shaded areas indicate 95% CIs.

Compared with NC, persons who were formerly incarcerated in RI experienced a sustained decrease of 157 opioid overdose deaths per 100 000 person-years (95% CI, −204 to −110 per 100 000 person-years) every 6 months after Medicaid expansion (Table 2; Figure 2A). Subgroup analyses results by race and ethnicity (Table 3, eFigure 3 in Supplement 1) and age (Table 3, eFigure 6 in Supplement 1) showed similar results as unintentional drug overdose deaths. Likewise, after Medicaid expansion implementation, people who were formerly incarcerated in RI experienced a sustained decrease of 62 polydrug overdose deaths per 100 000 person-years (95% CI, −96 to −28 per 100 000 person-years) every 6 months, compared with no expansion in NC (Table 2; Figure 2B). By the end of the study period, however, both NC and RI polydrug overdose mortality rates were increasing (Figure 2B).

### Suicide Deaths

Two-year postrelease suicide mortality rates among people who were formerly incarcerated remained relatively stable in both the RI and NC populations before Medicaid expansion (Table 1; Figure 1C). There was no substantial change after Medicaid expansion in suicide mortality rates among people who were formerly incarcerated in RI compared with NC (Table 2, Figure 1C).

### Homicide

Two-year postrelease homicide mortality rates slightly increased for both RI and NC (Table 1, Figure 1D). While NC mortality rates continued to increase at the same rate as pre-2014 rates, people who were formerly incarcerated in RI experienced a sustained decrease of 23 homicide deaths per 100 000 person-years (95% CI, −50 to 4 per 100 000 person-years) each year after the implementation of Medicaid expansion (Table 2; Figure 1D).

### Sensitivity Analysis

Sensitivity analyses showed larger decreases in sustained trend changes for all outcomes after accounting for potential delays in Medicaid expansion changes (eTable 5 in Supplement 1). Medicaid expansion was associated with an immediate and sustained decrease in reincarceration in RI compared with NC (eTable 6, eFigure 8 in Supplement 1). Furthermore, the all-cause mortality rates decreased mostly among individuals with a reincarceration history, but remained stable throughout the study period among individuals with only 1 incarceration (eTable 7 and eFigure 7 in Supplement 1).

## Discussion

In this study, which is, to our knowledge, the first to evaluate the association between Medicaid expansion and mortality in individuals who were formerly incarcerated, we found that all-cause, drug overdose, and homicide mortality rates decreased over time in RI after Medicaid expansion in January 2014 compared with NC. However, we found no substantial change in suicide mortality after Medicaid expansion. We also found that reincarceration rates decreased in RI following Medicaid expansion and all-cause mortality decreased most among individuals with reincarcerations, suggesting that reincarceration may modify the Medicaid expansion and mortality association. Although White individuals and those aged 30 years or older exhibited the highest mortality rates in 2009, these groups experienced the greatest decreases in trends after Medicaid expansion. However, all-cause mortality rates among Black individuals continued to increase after Medicaid expansion, and the combined group of racially minoritized individuals experienced modest decreases.

The largest cause-specific decrease was noted in unintentional drug overdose mortality from opioids by 157 fewer deaths (172 fewer deaths for all drug overdoses) per 100 000 person-years every 6 months after Medicaid expansion in RI compared with NC. While NC overdose death rates increased rapidly after 2014, mirroring US-wide trends attributed to fentanyl,^24^ RI overdose rates decreased after Medicaid implementation. Prior studies have reported that Medicaid expansion improves MOUD access.^12,13,25^ Ongoing studies are examining strategies for community-based MOUD linkage after release from prisons and jails.^26^ However, care linkages without insurance may remain unsustainable.^7^ Prerelease Medicaid enrollment in Wisconsin increased postrelease outpatient OUD care visits.^18^ Our study extends this work further, observing sustained decreases in drug overdose death rates each year between 2014 and 2018. Furthermore, buoyed by Medicaid expansion, by January 2017, RI had implemented a statewide program to provide MOUD in all carceral facilities^16^ that resulted in 60% reduction in opioid overdose mortality rates among persons who were formerly incarcerated from 2016 to 2017.^16^ We also saw that sharp reduction of overdose death rates from 2016 to 2017 in addition to the already decreasing rates after Medicaid expansion. However, rates returned to 2016 levels by the end of 2019 (Figure 2A), likely due to increase rise in polydrug overdoses in this population (Figure 2B), a trend mirrored across the US. Yet, there was a substantial overall decrease in overdose mortality after Medicaid expansion.

However, we observed, with respect to the outcomes studied, the benefits of Medicaid were experienced by White individuals, while Black individuals experienced negligible benefits. A similar racialized disparity was observed in the Wisconsin study, where prerelease Medicaid enrollment was associated with a larger increase in OUD care outpatient visits among White individuals than Black individuals.^18^ Similarly, the benefit of the RI statewide program to provide MOUD in all carceral facilities was largely experienced by White individuals and not among racially minoritized people.^16^ While large-scale structural policy change is important to improve population-level outcomes, racialized disparities in which population experiences the policy’s benefits is concerning given that overdose death rates are rapidly increasing among racially minoritized individuals in the US.^27^ Factors such as lack of resources to enroll in and access care through Medicaid, segregation, and medical mistrust fueled by implicit and explicit racialized biases in health care delivery may limit MOUD and health care access for racially minoritized people.^28,29^ Community-based, peer-led programs may be helpful in facilitating enduring linkage to care for people who were formerly incarcerated. Qualitative research may help identify strategies to best implement Medicaid expansion and similar policy initiatives tailored to specific community needs and for people who are racially minoritized.

In this study, we also observed a reduction in postrelease homicide mortality after Medicaid expansion in RI. Another study found a similar impact of Medicaid expansion in the general population.^30^ Increased health care access may increase resilience to mental and emotional trauma from loss of family connections, help persons who were formerly incarcerated better reintegrate and contribute to society,^4,7,11,12,13,31^ increase their ability to obtain employment, and steer them away from destabilizing high-risk or criminal activities, thereby preventing mortality from homicide.

Similarly, Medicaid expansion has been observed to increase access to mental health care and reduce suicide mortality in the general population.^15,32^ However, we did not see any change in suicide mortality among people who were formerly incarcerated. Our results are supported by another study that found increased health insurance coverage among individuals with criminal legal involvement after Medicaid expansion, but no corresponding increase in access to mental health care.^33^

### Limitations

We note some limitations that are tempered by corresponding strengths of the data and study design. First, we lack death data for released individuals who moved out of state, which potentially underestimates the rate in both states. However, most people are released on probation or parole of 9 months to a year, which reduces the underestimation probability of 1-year postrelease mortality rates. Second, RI and NC data may not be representative of people who were formerly incarcerated in all Medicaid expansion and nonexpansion states. However, pre-Medicaid expansion mortality rates in RI trended similar to those in NC, a state that trends similar to the rest of the country. Third, given the small study population size of RI, we were not able to fully examine EMM in the policy impact for all racial and ethnic groups. However, the evidence of racialized disparities is robust in our sensitivity analyses and is supported by prior studies.^16,18^ Fourth, the impact of Medicaid expansion may not be fully idealized in some chronic health conditions that respond to treatment and care access gradually.

## Conclusions

In this quasi-experimental cohort study, Medicaid expansion was associated with reductions in postrelease mortality among people who were formerly incarcerated, specifically from drug overdose and homicides. However, there were large, racialized inequities in the impact of Medicaid expansion. Future studies should focus on strategies to engage racially minoritized people who were formerly incarcerated in accessing health care and preventing harm.

## Notes

Supplement 1. **eMethods 1.** The Statistical ARIMA Model **eTable 1.** Causes of Death Examined in This Study Among CJ Involved Individuals From RI and NC, 2007-2019 **eTable 2.** Release-Level Demographic Distributions of Formerly Incarcerated People in Rhode Island and North Carolina by Medicaid Expansion Time Period, 2009-2018 **eTable 3.** Mortality Rates per 100 000 Person Years Among Formerly Incarcerated People by Race and Ethnicity, and Medicaid Expansion Period in Rhode Island and North Carolina, 2009-2018 **eTable 4.** Mortality Rates per 100 000 Person Years Among Formerly Incarcerated People by Race and Ethnicity, Sex, and Age and Medicaid Expansion Period in Rhode Island and North Carolina, 2009-2018 **eMethods 2.** Sensitivity Analyses **eTable 5.** Sensitivity Analysis Examining Potential Delayed Impact of Medicaid Expansion Implementation on Study Results **eTable 6.** Immediate and Sustained Changes in Reincarceration Rates Within One Year Post Release After Medicaid Expansion Implementation in 2014 in Rhode Island Relative to North Carolina, 2009-2018 **eTable 7.** Immediate and Sustained Changes in One-Year All-Cause Mortality Rates Within One Year Post Release After Medicaid Expansion Implementation in 2014 in Rhode Island Relative to North Carolina, 2009-2018 **eFigure 1.** All-Cause Mortality ITS Graphs by Race and Ethnicity **eFigure 2.** Unintentional Drug Overdose ITS Graphs by Race and Ethnicity **eFigure 3.** Unintentional Opioid Overdose ITS Graphs by Race and Ethnicity **eFigure 4.** All-Cause Mortality ITS Graphs by Age **eFigure 5.** Unintentional Drug Overdose ITS Graphs by Age **eFigure 6.** Unintentional Opioid Overdose ITS Graphs by Age **eFigure 7.** All-Cause Mortality ITS Graphs by Reincarceration Status **eFigure 8.** Reincarceration Within One Year After Release ITS Graph

Supplement 2.
Data Sharing Statement
