# Supplementary material for: Medicaid Expansion and Mortality Among Persons Who Were Formerly Incarcerated
Source: JAMA Netw Open. 2024 Sep 17;7(9):e2429454. doi: 10.1001/jamanetworkopen.2024.29454 (PMC11409152; doi:10.1001/jamanetworkopen.2024.29454)
Supplement: Supplement 1. — eMethods 1. The Statistical ARIMA Model eTable 1. Causes of Death Examined in This Study Among CJ Involved Individuals From RI and NC, 2007-2019 eTable 2. Release-Level Demographic Distributions of Formerly Incarcerated People in Rhode Island and North Carolina by Medicaid Expansion Time Period, 2009-2018 eTable 3. Mortality Rates per 100 000 Person Years Among Formerly Incarcerated People by Race and Ethnicity, and Medicaid Expansion Period in Rhode Island and North Carolina, 2009-2018 eTable 4. Mortality Rates per 100 000 Person Years Among Formerly Incarcerated People by Race and Ethnicity, Sex, and Age and Medicaid Expansion Period in Rhode Island and North Carolina, 2009-2018 eMethods 2. Sensitivity Analyses eTable 5. Sensitivity Analysis Examining Potential Delayed Impact of Medicaid Expansion Implementation on Study Results eTable 6. Immediate and Sustained Changes in Reincarceration Rates Within One Year Post Release After Medicaid Expansion Implementation in 2014 in Rhode Island Relative to North Carolina, 2009-2018 eTable 7. Immediate and Sustained Changes in One-Year All-Cause Mortality Rates Within One Year Post Release After Medicaid Expansion Implementation in 2014 in Rhode Island Relative to North Carolina, 2009-2018 eFigure 1. All-Cause Mortality ITS Graphs by Race and Ethnicity eFigure 2. Unintentional Drug Overdose ITS Graphs by Race and Ethnicity eFigure 3. Unintentional Opioid Overdose ITS Graphs by Race and Ethnicity eFigure 4. All-Cause Mortality ITS Graphs by Age eFigure 5. Unintentional Drug Overdose ITS Graphs by Age eFigure 6. Unintentional Opioid Overdose ITS Graphs by Age eFigure 7. All-Cause Mortality ITS Graphs by Reincarceration Status eFigure 8. Reincarceration Within One Year After Release ITS Graph [file jamanetwopen-e2429454-s001.pdf]

Supplementary Online Content

Perera PS, Miller VE, Fitch KV, et al. Medicaid expansion and postrelease mortality among persons who were formerly incarcerated. *JAMA Netw Open*. 2024;7(9):e2429454. doi:10.1001/jamanetworkopen.2024.29454

eMethods 1. The Statistical ARIMA Model

eTable 1. Causes of Death Examined in This Study Among CJ Involved Individuals From RI and NC, 2007-2019

eTable 2. Release-Level Demographic Distributions of Formerly Incarcerated People in Rhode Island and North Carolina by Medicaid Expansion Time Period, 2009-2018

eTable 3. Mortality Rates per 100,000 Person Years Among Formerly Incarcerated People by Race and Ethnicity, and Medicaid Expansion Period in Rhode Island and North Carolina, 2009-2018

eTable 4. Mortality Rates per 100,000 Person Years Among Formerly Incarcerated People by Race and Ethnicity, Sex, and Age and Medicaid Expansion Period in Rhode Island and North Carolina, 2009-2018

eMethods 2. Sensitivity Analyses

eTable 5: Sensitivity Analysis Examining Potential Delayed Impact of Medicaid Expansion Implementation on Study Results

eTable 6. Immediate and Sustained Changes in reincarceration Rates Within One Year Post Release After Medicaid Expansion Implementation in 2014 in Rhode Island Relative to North Carolina, 2009-2018.

eTable 7. Immediate and Sustained Changes in one-Year All-Cause Mortality Rates Within One Year Post Release After Medicaid expansion Implementation in 2014 in Rhode Island relative to North Carolina, 2009-2018

eFigure 1. All-Cause Mortality ITS Graphs by Race and Ethnicity

eFigure 2. Unintentional Drug Overdose ITS Graphs by Race and Ethnicity

eFigure 3. Unintentional Opioid Overdose ITS Graphs by Race and Ethnicity

eFigure 4. All-Cause Mortality ITS Graphs by Age

eFigure 5. Unintentional Drug Overdose ITS Graphs by Age

eFigure 6. Unintentional Opioid Overdose ITS Graphs by Age

eFigure 7. All-Cause Mortality ITS Graphs by Reincarceration Status

eFigure 8. Reincarceration Within One Year After Release ITS Graph

This supplementary material has been provided by the authors to give readers additional information about their work.

© 2024 Perera PS et al. *JAMA Network Open*.

eMethods 1. The statistical ARIMA model is as follows:

$$\lambda(T,X) = \beta_0 + \beta_1 T + \beta_2 I(T>T_{\text{ME}}) + \beta_3(T-T_{\text{ME}})*I(T>T_{\text{ME}}) + \beta_4 X + \beta_5 T*X + \beta_6 I(T>T_{\text{ME}})*X + \beta_7(T-T_{\text{ME}})*I(T>T_{\text{ME}})*X + \epsilon$$

Where, $\lambda(T,X)$ represents the morality rate per 100,000 person-years at time interval T (in quarters, 6-month period, or annual), for exposure group X (1=RI, 0=NC), and T_{ME} is the full time-interval after Medicaid expansion occurred, I is an indicator function that is 1 if the inside argument is true, 0 otherwise, and ϵ is the error term. And the coefficients,

β_0 = the baseline (at time 1) mortality rate (all-cause or cause-specific) among formerly incarcerated individuals in NC

β_1 = the pre-Medicaid expansion trend (slope function) of the mortality rates for NC

β_2 = the immediate absolute change at the first post-expansion time point (also known as level change) in mortality rates in NC after the Medicaid expansion was implemented in RI (we do not expect there to be any change here, since the Medicaid expansion was not implemented in NC and no other known policy change occurred during that time that will immediately affect formerly incarcerated individuals' health in NC);

β_3 = pre- and post-expansion trend change (slope functions) of the mortality rates in NC = NC post-expansion trend minus β_1;

β_4 = baseline mortality rate difference between RI and NC at time 1 = RI baseline mortality rate minus β_0

β_5 = pre-expansion mortality rate trend difference between RI and NC = RI pre-expansion mortality rate trend minus β_1;

β_6 = immediate absolute change in RI mortality rates relative to NC after the Medicaid expansion implementation = RI level change minus β_2;

β_7 = pre-post expansion mortality rates trend difference in RI relative to NC after Medicaid expansion = (RI post-trend – RI pre-trend) – β_3.

Of main interest to us are β_6 and β_7 that show the immediate and sustained impact of the Medicaid expansion, respectively, in RI while controlling for NC that we assume as a counterfactual for the national mortality rate trends among formerly incarcerated people.

© 2024 Perera PS et al. *JAMA Network Open*.

eTable 1. Causes of death examined in this study among CJ involved individuals from RI and NC, 2007-2019

Cause of Death	ICD 10 codes with subcategories
Unintentional drug overdose death[a]	X40-X44 underlying cause of deaths
Opioid overdose death[a]	X40-X44 underlying cause of death and/or T40.0 – T40.4, and T40.6 contributing cause of death
Polydrug overdose death[a]	Underlying cause of death X40-X44, including contributory codes for at least two of the following for an opioid (T40.0-T40.5, T40.6), cocaine (T40.5), methamphetamine (T43.6), and benzodiazepine (T42.4)
Suicide Death	X60–X84, Y87.0 underlying causes of death only
Homicide Death	X85–Y09, Y87.1 underlying causes of death only

a – intentional and undetermined intent overdose deaths were excluded.

© 2024 Perera PS et al. *JAMA Network Open*.

eTable 2. Release-level demographic distributions of formerly incarcerated people in Rhode Island and North Carolina by Medicaid expansion time period, 2009-2018.

Characteristic	All releases		Pre-Medicaid Expansion releases		Post-Medicaid Expansion releases	
	RI (N=35,350) N (%)	NC (N=238,781) N (%)	RI (N=18,806) N (%)	NC (N=125,147) N (%)	RI (N=16,544) N (%)	NC (N=113,634) N (%)
Race and Ethnicity						
Hispanic (any race)	6,554 (18.5)	4,416 (1.9)	3,286 (17.5)	2,610 (2.1)	3,268 (19.8)	1,806 (1.6)
Non-Hispanic American native	285 (0.8)	4,750 (2.0)	116 (0.6)	2,351 (1.9)	169 (1.0)	2,399 (2.1)
Non-Hispanic Asian or Pacific islander	298 (0.8)	535 (0.2)	161 (0.9)	268 (0.2)	137 (0.8)	267 (0.2)
Non-Hispanic black	8,854 (25.1)	121,221 (50.8)	4,720 (25.1)	65,902 (52.7)	4,314 (25.0)	55,319 (48.7)
Non-Hispanic white	19,026 (53.8)	102,082 (42.8)	10,412 (55.4)	50,793 (40.6)	8,614 (52.1)	51,289 (45.1)
Not categorized above[a]	333 (0.9)	5,777 (2.4)	111 (0.6)	3,223 (2.6)	222 (1.3)	2,554 (2.3)
Sex						
Female	3,838 (10.9)	29,760 (12.5)	2,003 (10.7)	15,043 (12.0)	1,835 (11.1)	14,717 (13.0)
Male	31,512 (89.1)	209,021 (87.5)	16,803 (89.4)	110,104 (88.0)	14,709 (88.9)	98,917 (87.1)
Age						
<30	14,447 (40.9)	97,034 (40.6)	7,898 (42.0)	51,210 (40.9)	6,549 (39.6)	45,824 (40.3)
30+	20,902 (59.1)	141,747 (59.4)	10,908 (58.0)	73,937 (59.1)	9,995 (60.4)	67,810 (59.7)

a. Not categorized above includes unknown, multiple races, or another race that was not reported.

© 2024 Perera PS et al. *JAMA Network Open*.

eTable 3. Mortality rates per 100,000 person years among formerly incarcerated people by race and ethnicity, and Medicaid expansion period in Rhode Island and North Carolina, 2009-2018.

	**Overall Rate**		**Pre-Medicaid Expansion**		**Post-Medicaid Expansion**	
	**RI Rate (95% CI)**	**NC Rate (95% CI)**	**RI Rate (95% CI)**	**NC Rate (95% CI)**	**RI Rate (95% CI)**	**NC Rate (95% CI)**
**All-Cause Mortality**						
Race and Ethnicity						
Hispanic (any race)	692 (487, 953)	440 (261, 695)	641 (373, 1,026)	199 (65, 465)	742 (453, 1,145)	822 (438,1,406)
Non-Hispanic American native	1345 (277, 3932)	933 (663, 1275)	3190 (658, 9327)	637 (348, 1069)	**0**	126 (816, 1862)
Non-Hispanic Asian or Pacific islander	401 (10, 2229)	**0**	718 (18, 4,008)	**0**	**0**	**0**
Non-Hispanic black	714 (528, 944)	590 (545, 637)	574 (355, 877)	473 (420, 531)	874 (581, 1263)	743 (667, 825)
Non-Hispanic white	1385 (1203, 1587)	1195 (1125, 1268)	1153 (933, 1409)	972 (885, 1064)	1668 (1375, 2005)	1534 (1419, 1655)
Not categorized above[a]	369 (9, 2056)	197 (99, 353)	1029 (26, 5744)	31 (1, 175)	**0**	418 (200, 769)
**Unintentional Drug Overdose**						
Race and Ethnicity						
Hispanic (any race)	430 (273, 645)	195 (84, 385)	377 (181, 693)	40 (1, 222)	482 (257, 824)	443 (178, 912)
Non-Hispanic American native	**0**	335 (183, 562)	**0**	**0**	**0**	706 (386, 1,185)
Non-Hispanic Asian or Pacific islander	**0**	**NA**	**0**	**0**	**0**	**0**
Non-Hispanic black	233 (133, 378)	74 (58, 92)	137 (44, 319)	24 (14, 40)	343 (171, 614)	138 (107,176)
Non-Hispanic white	826 (687, 985)	478 (434, 525)	607 (450, 800)	281 (236, 333)	1092 (858, 1371)	695 (619, 778)
Not categorized above[a]	**0**	18 (1, 100)	**0**	31 (1, 175)	**0**	**0**
**Opioid Overdose**						
Race and Ethnicity						
Hispanic (any race)	393 (243, 600)	195 (84, 385)	339 (155, 644)	40 (1, 222)	445 (82, 777)	443 (178, 912)
Non-Hispanic American native	**0**	311 (166, 532)	**0**	**0**	**0**	656 (349, 1,122)
Non-Hispanic Asian or Pacific islander	**0**	**0**	**0**	**0**	**0**	**0**

© 2024 Perera PS et al. *JAMA Network Open*.

	Overall Rate		Pre-Medicaid Expansion		Post-Medicaid Expansion	
	RI Rate (95% CI)	NC Rate (95% CI)	RI Rate (95% CI)	NC Rate (95% CI)	RI Rate (95% CI)	NC Rate (95% CI)
Non-Hispanic black	160 (80, 287)	56 (43, 72)	109 (30, 280)	15 (7, 28)	218 (88, 450)	111 (83,145)
Non-Hispanic white	759 (626, 912)	421 (380, 465)	546 (398, 731)	220 (180, 267)	1018 (792, 1289)	642 (569, 722)
Not categorized above[a]	**0**	18 (1, 100)	**0**	31 (1, 175)	**0**	**0**
**Polydrug Overdose**						
Race and Ethnicity						
Hispanic (any race)	187 (90, 344)	122 (40, 285)	188 (61, 440)	40 (1, 222)	185 (60, 433)	253 (69, 648)
Non-Hispanic American native	**0**	311 (166, 532)	**0**	**0**	**0**	454 (208, 862)
Non-Hispanic Asian or Pacific islander	**0**	**0**	**0**	**0**	**0**	**0**
Non-Hispanic black	87 (32, 190)	33 (23, 46)	82 (17, 239)	6 (2, 17)	94 (19, 273)	68 (47, 96)
Non-Hispanic white	479 (375, 604)	199 (171, 231)	376 (256, 534)	73 (51,102)	605 (434, 821)	338 (286, 398)
Not categorized above[a]	**0**	**0**	**0**	**0**	**0**	**0**
**Suicide**						
Race and Ethnicity						
Hispanic (any race)	11 (0, 61)	51 (14, 132)	22 (1, 120)	41 (5,149)	**0**	68 (8, 247)
Non-Hispanic American native	**0**	**0**	**0**	**0**	**0**	**0**
Non-Hispanic Asian or Pacific islander	**0**	**0**	**0**	**0**	**0**	**0**
Non-Hispanic black	44 (14,102)	15 (10, 21)	64 (18, 165)	14 (8, 23)	19 (0, 106)	15 (8, 26)
Non-Hispanic white	104 (69,152)	81 (66, 109)	90 (48,154)	73 (56, 93)	122 (67, 205)	90 (70, 114)
Not categorized above[a]	215 (5, 1198)	28 (6, 81)	575 (15, 3202)	**0**	**0**	65 (13, 190)
**Homicide**						
Race and Ethnicity						
Hispanic (any race)	120 (60, 214)	39 (8,113)	151 (61, 311)	21 (1,115)	88 (24, 225)	68 (8, 247)

© 2024 Perera PS et al. *JAMA Network Open*.

	Overall Rate		Pre-Medicaid Expansion		Post-Medicaid Expansion	
	**RI Rate (95% CI)**	**NC Rate (95% CI)**	**RI Rate (95% CI)**	**NC Rate (95% CI)**	**RI Rate (95% CI)**	**NC Rate (95% CI)**
Non-Hispanic American native	**0**	212 (121, 344)	**0**	197 (85, 387)	**0**	230 (99, 453)
Non-Hispanic Asian or Pacific islander	**0**	**0**	**0**	**0**	**0**	**0**
Non-Hispanic black	131 (73, 216)	200 (181 ,221)	145 (66, 275)	157 (135, 182)	114 (42, 248)	259 (226, 296)
Non-Hispanic white	35 (16, 66)	54 (43, 66)	28 (8, 71)	61 (46, 79)	44 (14,102)	46 (32, 63)
Not categorized above[a]	**0**	28 (6, 80)	**0**	16 (0, 89)	**0**	43 (5,157)

a. Not categorized above includes unknown, multiple races, or another race that was not reported.
American native: For RI there was a total of 3 deaths.
Asian: 1 death total for RI, 0 deaths for NC

© 2024 Perera PS et al. *JAMA Network Open*.

eTable 4. Mortality rates per 100,000 person years among formerly incarcerated people by race and ethnicity, sex, and age and Medicaid expansion period in Rhode Island and North Carolina, 2009-2018.

	**Overall Rate**		**Pre-Medicaid Expansion**		**Post-Medicaid Expansion**	
	**RI Rate (95% CI)**	**NC Rate (95% CI)**	**RI Rate (95% CI)**	**NC Rate (95% CI)**	**RI Rate (95% CI)**	**NC Rate (95% CI)**
**All-Cause Mortality**						
Race and Ethnicity						
Non-Hispanic black	714 (528, 944)	590 (545, 637)	574 (355, 877)	473 (420, 531)	874 (581,1263)	743 (667, 825)
Non-Hispanic white	1385 (1203, 1587)	1195 (1125, 1268)	1153 (933, 1409)	972 (885, 1064)	1,668 (1375, 2005)	1534 (1419, 1655)
All racially minoritized people combined	689 (497, 932)	474 (368, 601)	737 (462, 1116)	246 (150, 379)	643 (393, 993)	774 (571, 1,027)
Sex						
Female	1,144 (801,1,584)	779 (678, 892)	1335 (837, 2021)	512 (402, 643)	935 (511, 1568)	1,082 (909,1,278)
Male	1,059 (935, 1,195)	848 (806, 891)	876 (724, 1051)	680 (631,732)	1268 (1072, 1491)	1,054 (986, 1126)
Age						
<30	653 (512, 821)	596 (545, 650)	603 (424, 831)	460 (401, 525)	714 (500, 989)	769 (683,864)
30+	1346 (1176,1533)	999 (945, 1055)	1155 (94, 1403)	795 (730, 864)	1553 (1293, 1851)	1241 (1152, 1334)
**Unintentional Drug Overdose**						
Race and Ethnicity						
Non-Hispanic white	826 (687, 985)	478 (434, 525)	607 (450, 800)	281 (236, 333)	1092 (858, 1371)	695 (619, 778)
All racially minoritized people combined	301 (214, 411)	84 (68, 102)	226 (126, 372)	24 (14, 39)	380 (244, 566)	162 (129, 200)
Sex						
Female	667 (413, 1020)	338 (273, 415)	728 (376, 1272)	132 (79, 205)	601 (275, 1141)	572 (449, 720)
Male	572 (482, 674)	238 (217, 262)	400 (300, 524)	128 (107, 152)	768 (617, 945)	375 (334, 419)
Age						
<30	295 (203, 415)	179 (152, 210)	212 (113, 362)	90 (65, 122)	397 (242, 613)	292 (240, 353)

© 2024 Perera PS et al. *JAMA Network Open*.

	Overall Rate		Pre-Medicaid Expansion		Post-Medicaid Expansion	
	**RI Rate (95% CI)**	**NC Rate (95% CI)**	**RI Rate (95% CI)**	**NC Rate (95% CI)**	**RI Rate (95% CI)**	**NC Rate (95% CI)**
30+	774 (647, 919)	299 (270, 330)	594 (444, 780)	154 (127, 187)	969 (766, 1210)	469 (416, 528)
**Opioid Overdose**						
Race and Ethnicity						
Non-Hispanic white	759 (626, 912)	421 (380, 465)	546 (398, 731)	220 (180, 267)	1,018 (792, 1289)	642 (569, 722)
All racially minoritized people combined	247 (169, 349)	67 (54, 84)	196 (104, 335)	16 (8, 28)	301 (181, 470)	135 (106,171)
Sex						
Female	636 (388, 982)	305 (243, 378)	728 (376, 1272)	104 (58, 171)	534 (231, 1052)	533 (414, 676)
Male	507 (423, 604)	207 (185, 226)	347 (254, 464)	98 (80, 119)	690 (547, 859)	336 (298, 378)
Age						
<30	268 (181, 383)	158 (132, 187)	179 (89, 321)	76 (53, 105)	377 (227, 589)	263 (213, 320)
30+	691 (571, 828)	257 (230, 286)	537 (395, 715)	114 (91, 142)	857 (667, 1085)	425 (374, 481)
**Polydrug Overdose**						
Race and Ethnicity						
All racially minoritized people combined	123 (71, 201)	41 (30, 54)	120 (52, 237)	7 (2,17)	127 (55, 250)	85 (62, 113)
Non-Hispanic white	479 (375, 604)	199 (171, 231)	376 (256, 534)	73 (51, 102)	605 (434, 821)	338. (286, 398)
Sex						
Female	540 (315, 865)	199 (149, 259)	607 (291, 1116)	42 (15, 90)	467 (188, 963)	376 (277, 499)
Male	286 (223, 361)	95 (81, 110)	219 (147, 315)	33 (23, 46)	362 (261, 490)	171 (144, 202)
Age						
<30	134 (75, 221)	87 (68, 109)	98 (36, 213)	29 (16, 49)	179 (82, 339)	161 (123, 207)
30+	435 (341, 547)	122 (104, 142)	377 (260, 530)	37 (24, 54)	497 (355, 677)	222 (186, 263)
**Suicide**						
Race and Ethnicity						
Non-Hispanic white	104 (69, 152)	81 (16, 66)	90 (48, 154)	73 (57, 93)	122 (69, 205)	90 (70, 114)

© 2024 Perera PS et al. *JAMA Network Open*.

	Overall Rate		Pre-Medicaid Expansion		Post-Medicaid Expansion	
	**RI Rate (95% CI)**	**NC Rate (95% CI)**	**RI Rate (95% CI)**	**NC Rate (95% CI)**	**RI Rate (95% CI)**	**NC Rate (95% CI)**
All racially minoritized people combined	54 (22, 111)	29 (21, 41)	53 (19, 114)	14 (8, 22)	10 (0, 53)	19 (11, 30)
Sex						
Female	72 (20, 185)	28 (15, 46)	67 (8, 242)	18 (6, 43)	78 (9, 282)	39 (18,74)
Male	71 (48, 101)	46 (39, 54)	74 (43, 119)	41 (32 ,51)	67 (36, 115)	52 (41, 65)
Age						
<30	74 (41, 125)	46 (36, 58)	86 (39, 162)	39 (27, 54)	60 (20, 141)	56 (39, 77)
30+	69 (42, 106)	42 (34, 51)	65 (31, 120)	37 (28, 49)	73 (35, 135)	47 (35, 62)
**Homicide**						
Race and Ethnicity						
Non-Hispanic white	35 (16, 66)	54 (43, 66)	28 (8, 71)	61 (46, 79)	44 (14, 102)	46 (32, 63)
All racially minoritized people combined	201 (131, 294)	341 (310, 376)	140 (80, 227)	146 (126, 168)	95 (46, 175)	241 (211, 274)
Sex						
Female	54 (11,158)	29 (17, 49)	67 (8, 242)	25 (10, 52)	39 (1, 217)	34 (15, 68)
Male	76 (52, 107)	145 (133, 158)	79 (47, 124)	124 (109, 141)	72 (39, 121)	172 (152, 195)
Age						
<30	96 (57, 151)	194 (173, 218)	95 (46, 175)	170 (144, 200)	96 (42, 190)	227 (192, 267)
30+	59 (34, 94)	89 (34, 102)	65 (31, 120)	72 (58, 88)	51 (21, 106)	110 (91, 131)

© 2024 Perera PS et al. *JAMA Network Open*.

eMethods 2. Sensitivity analyses:

We conducted sensitivity analyses by moving the effective policy implementation date from January 2014 to later dates to reflect delayed effects of policy implementation (additional details in supplemental materials). For all-cause mortality, we calculated 1-year post-release mortality rates for each quarter of a calendar year, then CITS analysis was conducted by simulating the delay of Medicaid expansion effect by three months, six months, and one year. For all unintentional overdose mortality, we calculated 1-year post-release mortality rates for each six-month interval of a calendar year, sensitivity analysis was then conducted by delaying the Medicaid expansion effect by six months and one year. And for homicide and suicide mortality, we calculated 2-year post-release mortality rates for each calendar year, sensitivity analysis was then conducted by delaying the Medicaid expansion effect by one year.

© 2024 Perera PS et al. *JAMA Network Open*.

eTable 5: Sensitivity analysis examining potential delayed impact of Medicaid Expansion implementation on study results

**Dates representing actual and delayed implementation of Medicaid Expansion**	**Immediate absolute change in mortality rate after Medicaid expansion β_6 (95% CI)**	**Sustained trend change in mortality rate per quarter after Medicaid expansion β_7 (95% CI)**
All-Cause Mortality [a,c]		
January 1st, 2014	280 (-135, 695)	-72 (-108, -36)
April 1st, 2014 (3 months delay)	60 (-363, 483)	-75 (-112, -38)
July 1st, 2014 (6 months delay)	219 (-198, 636)	-80 (-118, -43)
January 1st, 2015 (1 year delay)	223 (-190, 635)	-93 (-133, -53)
Unintentional Drug Overdose [a,d]		
January 1st, 2014	74 (-240, 388)	-172 (-226, -118)
July 1st, 2014 (6 months delay)	-7 (-313, 299)	-180 (-235, -125)
January 1st, 2015 (1 year delay)	-282 (-583, 19)	-172 (-231, -112)
Unintentional Opioid Overdose [a,d]		
January 1st, 2014	45.37 (-226, 317)	-157 (-204, -110)
July 1st, 2014 (6 months delay)	25 (-233, 284)	-167 (-213, -120)
January 1st, 2015 (1 year delay)	-226 (-483, 30)	-161 (-212, -111)
Unintentional Polydrug Overdose [a,d]		
January 1st, 2014	-155 (-350, 40)	-62 (-96, -28)
July 1st, 2014 (6 months delay)	-167 (-366, 31)	-55(-90, -19)
January 1st, 2015 (1 year delay)	-184 (-387, -19)	-47 (-87, -7)
Suicide [b,e]		
July 1st, 2014 (6 months delay)	1 (-61, 64)	5 (-17, 26)
January 1st, 2015 (1 year delay)	-17 (-75, 41)	9 (-14, 33)
Homicide [b,e]		
January 1st, 2014	-9 (-88, 70)	-23 (-50, 4)
January 1st, 2015 (1 year delay)	5 (-66, 75)	-29 (-57, 0)

[a] One-year death rate per 100,000 person-years.
[b] Two-year death rate per 100,000 person-years.
[c] Trends calculated per quarter.
[d] Trends calculated per 6-month intervals.
[e] Trends calculated per year.

© 2024 Perera PS et al. *JAMA Network Open*.

eTable 6. Immediate and sustained changes in reincarceration rates within one year post release after Medicaid expansion implementation in 2014 in Rhode Island relative to North Carolina, 2009-2018.

**Outcome**	**Immediate absolute change in mortality rate after Medicaid expansion**<br>**β_6 (95% CI)**	**Sustained trend change in mortality rate after Medicaid expansion**<br>**β_7 (95% CI)**
Reincarceration within one year post release	-109 (-149, -68)	-2 (-4, -1)

© 2024 Perera PS et al. *JAMA Network Open*.

eTable 7. Immediate and sustained changes in one-year all-cause mortality rates within one year post release after Medicaid expansion implementation in 2014 in Rhode Island relative to North Carolina, 2009-2018.

**Outcome**	**Immediate absolute change in mortality rate after Medicaid expansion β_6 (95% CI)**	**Sustained trend change in mortality rate after Medicaid expansion β_7 (95% CI)**
No reincarceration within one year post release	372 (-226, 970)	-26 (-232, 179)
Reincarceration within one year post release	-117 (-611, 376)	-442 (-612, -273

© 2024 Perera PS et al. *JAMA Network Open*.

eFigure 1. All-cause mortality ITS graphs by race and ethnicity (not non-Hispanic white includes all racially minoritized people combined).

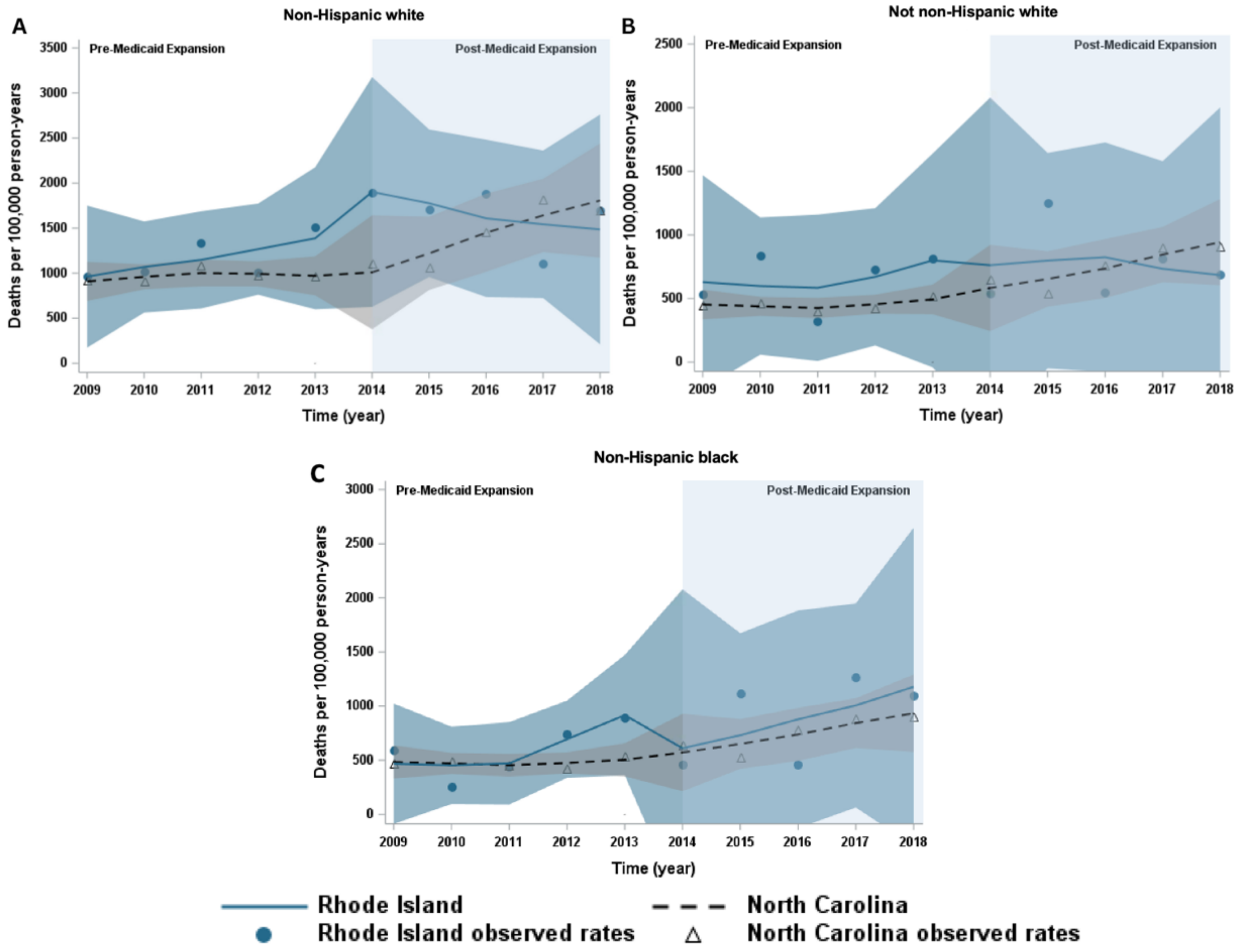

© 2024 Perera PS et al. *JAMA Network Open*.

eFigure 2. Unintentional drug overdose ITS graphs by race and ethnicity (not non-Hispanic white includes all racially minoritized people combined).

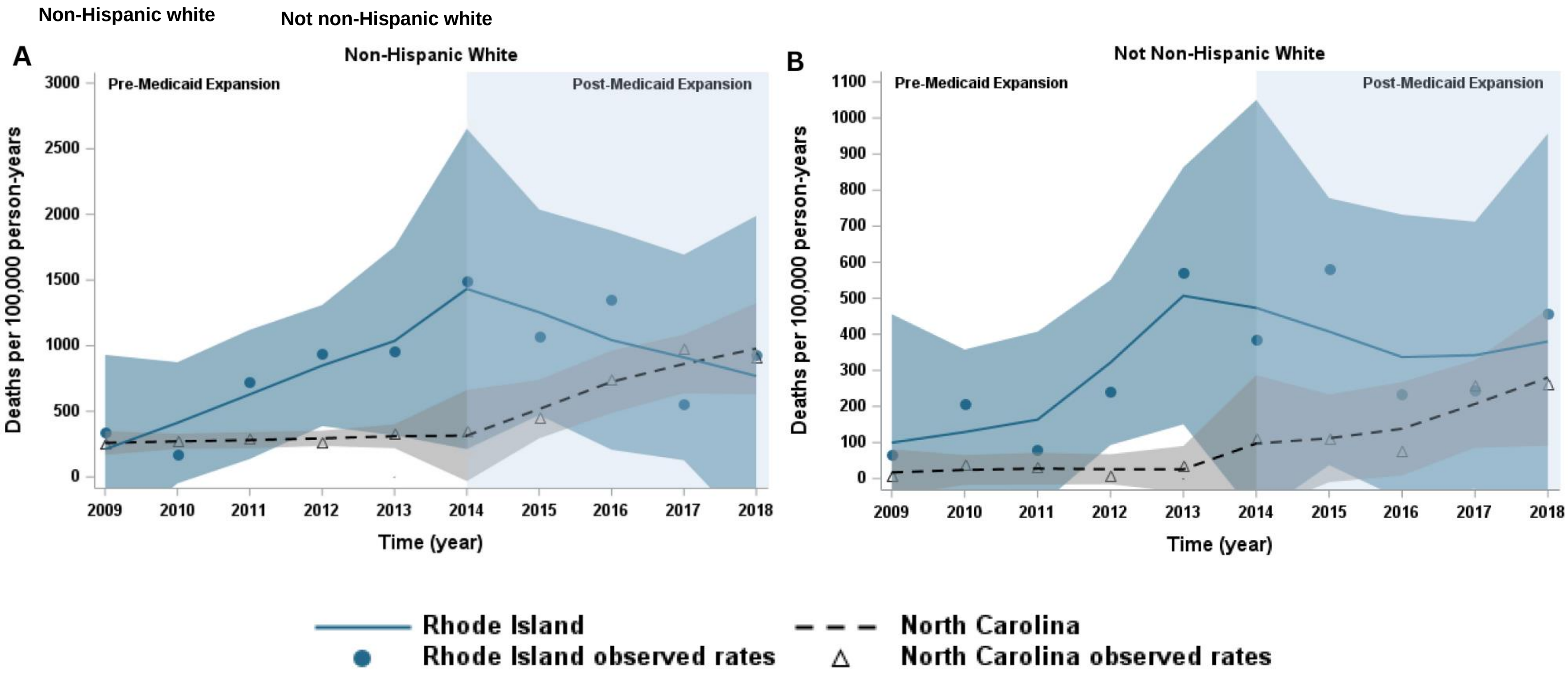

© 2024 Perera PS et al. *JAMA Network Open*.

eFigure 3. Unintentional opioid overdose ITS graphs by race and ethnicity (not non-Hispanic white includes all racially minoritized people combined).

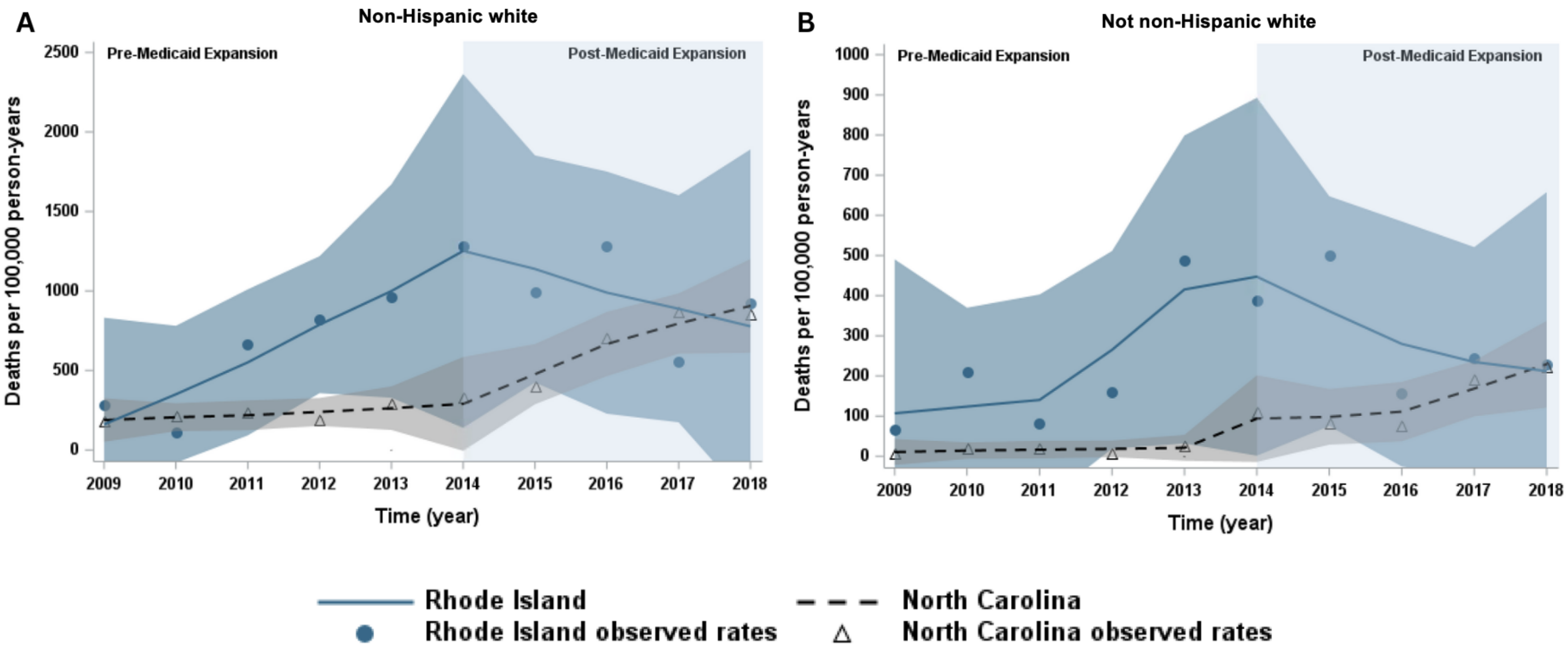

© 2024 Perera PS et al. *JAMA Network Open*.

eFigure 4. All-cause mortality ITS graphs by age.

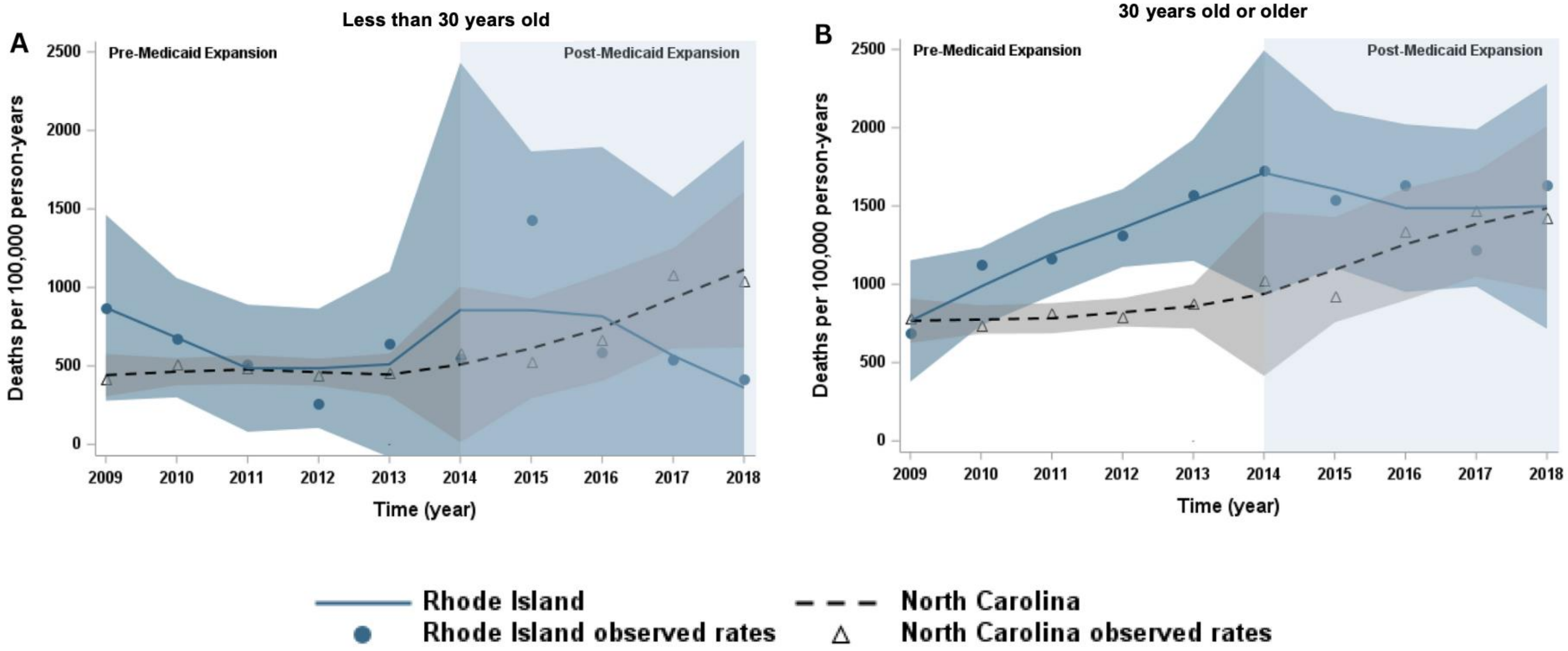

© 2024 Perera PS et al. *JAMA Network Open*.

eFigure 5. Unintentional drug overdose ITS graphs by age.

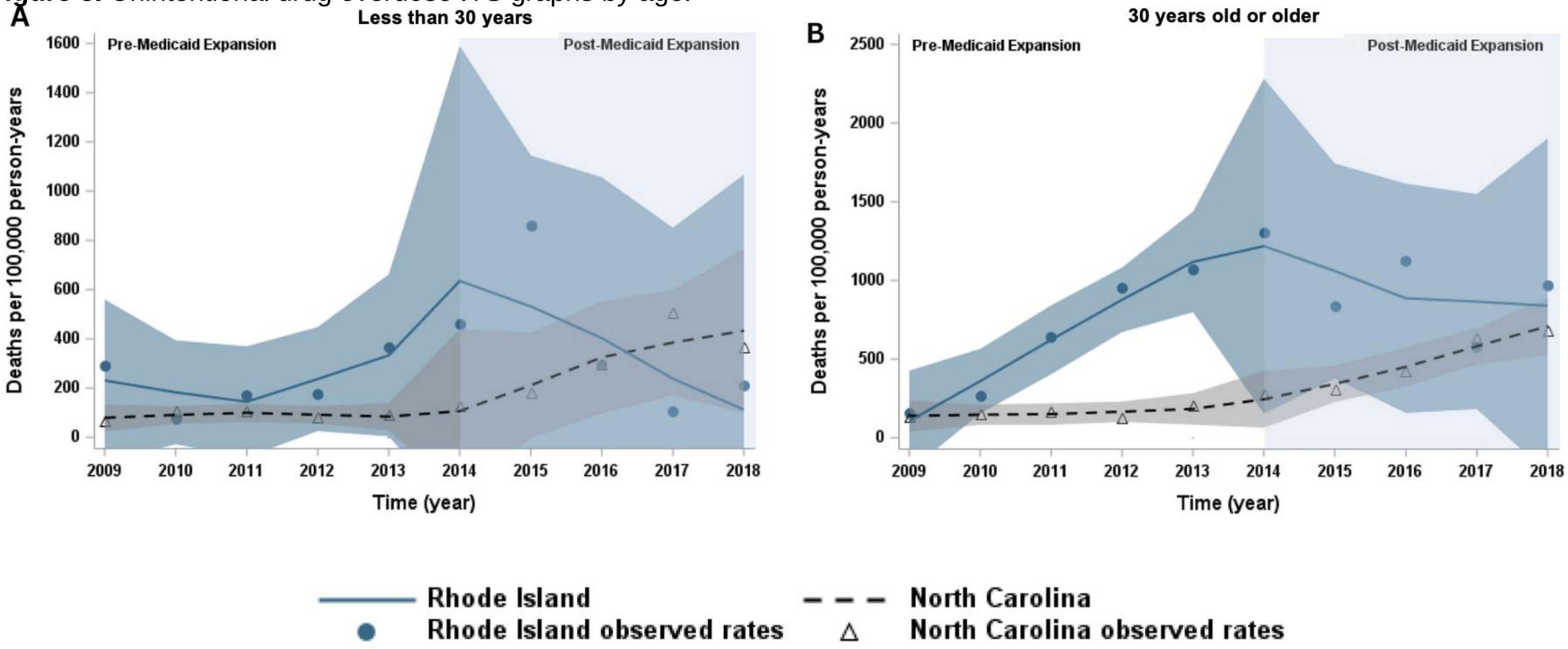

© 2024 Perera PS et al. *JAMA Network Open*.

eFigure 6. Unintentional opioid overdose ITS graphs by age.

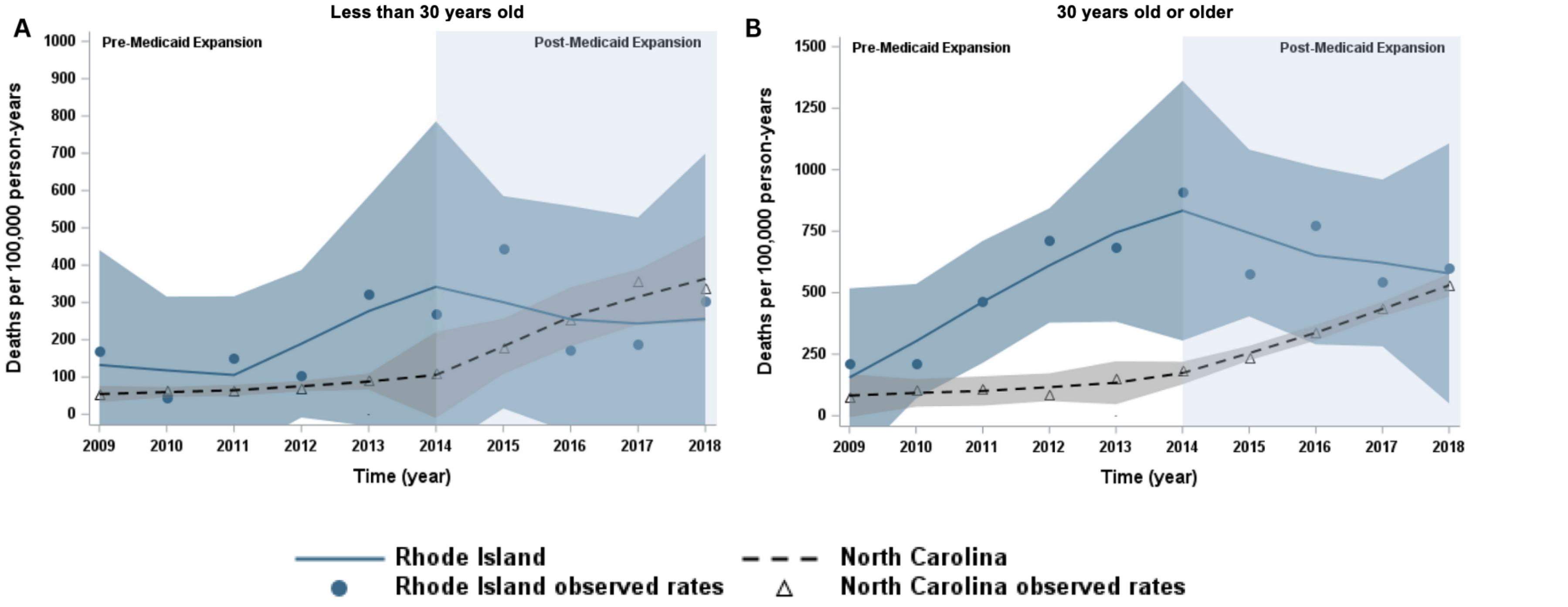

© 2024 Perera PS et al. *JAMA Network Open*.

eFigure 7. All-cause mortality ITS graphs by reincarceration status

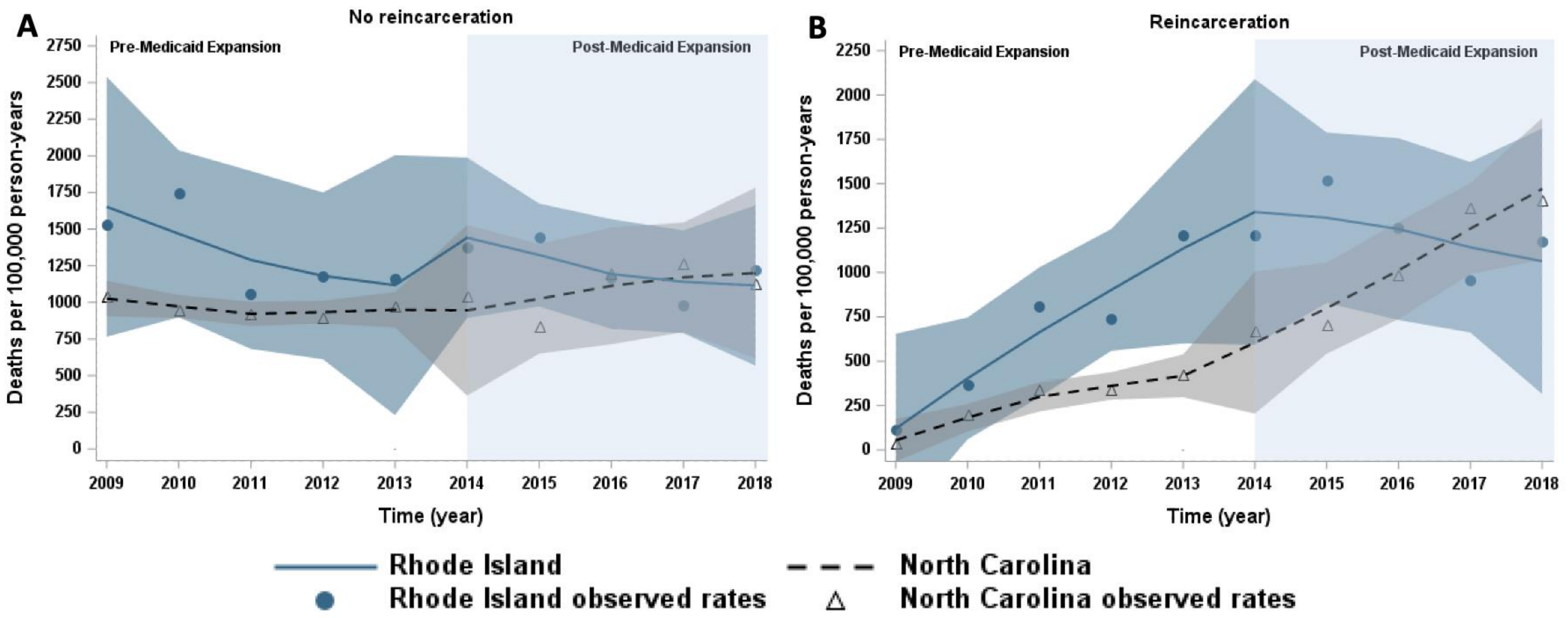

© 2024 Perera PS et al. *JAMA Network Open*.

eFigure 8. Reincarceration within one year after release ITS graph.

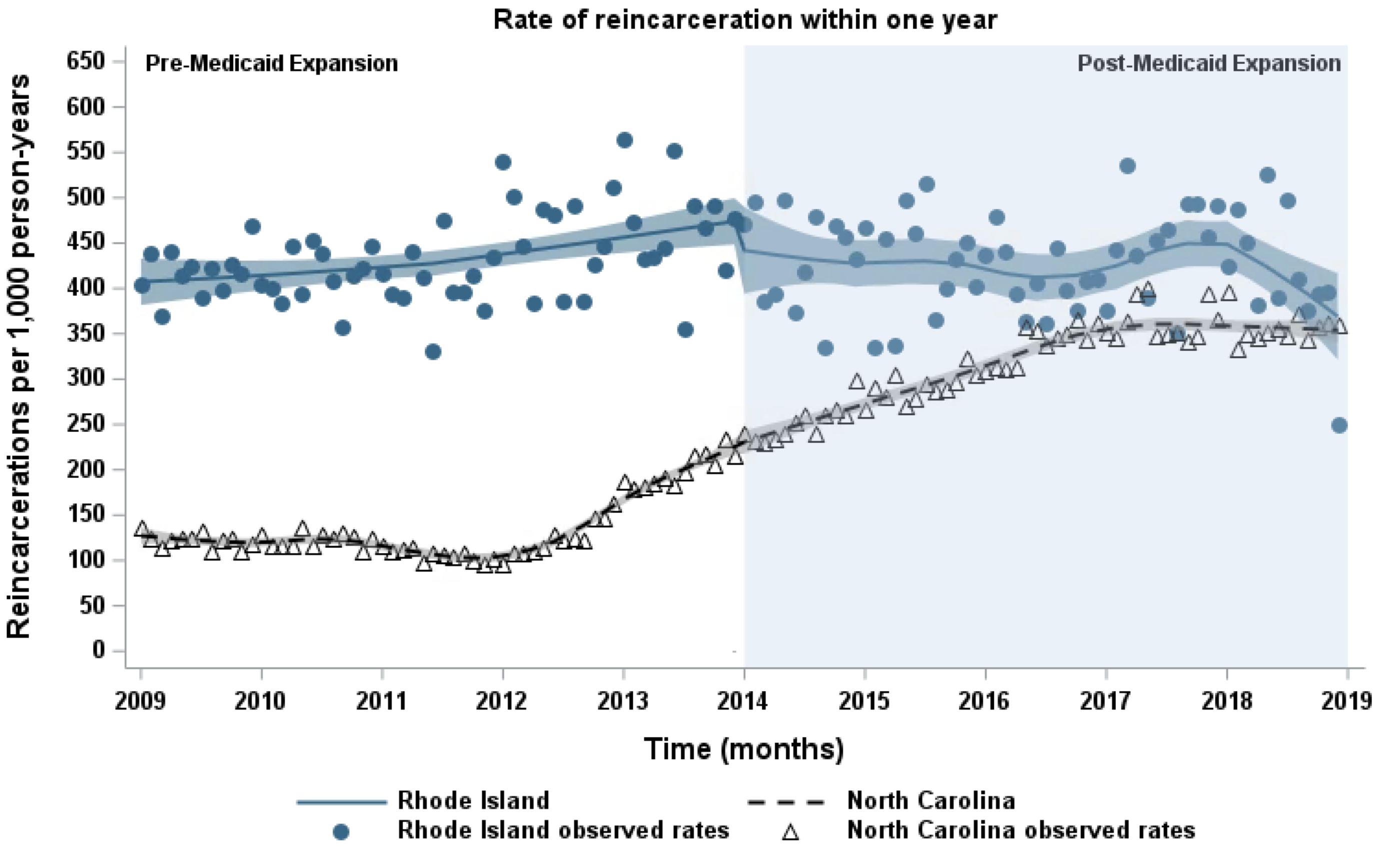

© 2024 Perera PS et al. *JAMA Network Open*.
