# Supplementary material for: Medicaid Expansion and Mortality Among Persons Who Were Formerly Incarcerated
Source: JAMA Netw Open. 2024 Sep 17;7(9):e2429454. doi: 10.1001/jamanetworkopen.2024.29454 (PMC11409152; doi:10.1001/jamanetworkopen.2024.29454)
Supplement: Supplement 2. — Data Sharing Statement [file jamanetwopen-e2429454-s002.pdf]

Data Sharing Statement

Perera. Medicaid Expansion and Mortality Among Persons Who Were Formerly Incarcerated. *JAMA Netw Open*. Published September 17, 2024. doi:10.1001/jamanetworkopen.2024.29454

Data
Data available: No

Additional Information
Explanation for why data not available: The data are protected by Data Use Agreements with the NC Department of Adult Corrections, RI Department of Health, and RI Department of Corrections. The data can be available upon request to these entities, and once approved, the authors of the study can share these data.
